# Synovial plica of the elbow — detailed measurements and how to implicate its relevance in clinical practice

**DOI:** 10.1007/s00264-023-05726-9

**Published:** 2023-02-21

**Authors:** Michał Bonczar, Patryk Ostrowski, Wojciech Bednarz, Wadim Wojciechowski, Jerzy Walocha, Mateusz Koziej

**Affiliations:** 1 Department of Anatomy, Jagiellonian University Medical College, Mikołaja Kopernika 12, 33-332 Cracow, Poland; 2 Youthoria, Youth Research Organization, Cracow, Poland; 3 Department of Radiology, Jagiellonian University Medical College, Cracow, Poland

**Keywords:** Elbow synovial fold, Plica, Lateral elbow, Radiohumeral joint, Elbow pain, Elbow synovial fold syndrome

## Abstract

**Purpose:**

The synovial plica of the elbow is a fold of synovial tissue, which is said to be a remnant of the embryonic septa of normal articular development and is located around the radiocapitellar joint. The objective of the present study was to provide morphometric properties of the synovial plica of the elbow and its relation to surrounding structures in asymptomatic patients.

**Methods:**

A retrospective study was conducted to establish the morphometric characteristics of the synovial plica of the elbow. The results of 216 consecutive patients, who for different reasons during the five year period of time underwent magnetic resonance imaging (MRI) of an elbow, were analyzed.

**Results:**

Plica was found in a total of 161 of 216 elbows (74.5%). The mean width of the plica was set to be 3.00 mm (SD: 1.39). The mean length of the plica was established at 2.91 mm (SD: 1.13). An analysis of sexual dimorphism was also included. Potential correlations were analyzed for each of the categories and age.

**Conclusions:**

The synovial plica of the elbow is a clinically relevant anatomical structure. Analyzing the morphometric parameters of the synovial plica is necessary to properly evaluate synovial plica syndrome, which can commonly be confused with other sources of lateral elbow pain such as tennis elbow, oppression of the radial and/or posterior interosseous nerve, or snapping of the triceps tendon. The authors suggest that the thickness of the plica may not be the golden diagnostic feature as there are no statistically significant differences in this parameter between symptomatic and asymptomatic patients. A precise and accurate diagnosis of synovial fold syndrome and/or differentiation from other sources of lateral elbow pain must be performed, as the surgical treatment, even if performed properly, will be unsuccessful because of a misdiagnosed source of pain.

## Introduction

The synovial plica of the elbow is a fold of synovial tissue, which is said to be a remnant of the embryonic septa of normal articular development and is located around the radiocapitellar joint [1]. Histologically, the plica is predominantly composed of fibroadipose tissue, with vascular networks and abundant nerve endings in the periphery [2]. Therefore, it cannot be classified as a meniscus due to its lack of fibrocartilage [1, 2]. The functional importance of the plica in asymptomatic patients is not fully understood [3]. Some studies suggest that it might act as a stabilizer to prevent excessive movement [3].

Pathologies of the plicae become symptomatic in people with chronic inflammation caused by repetitive athletic activities, such as throwing, or direct trauma [4]. Chronically inflamed plicae turn subsequently into fibrotic tissue folds that can cause posterolateral impingement or synovial plica syndrome [3]. Synovial plica syndrome is generally characterized by various symptoms, such as clicking or snapping during elbow motion, or pain on the lateral side of the elbow [4]. The initial treatment of the said syndrome is generally with conservative therapy. However, surgical treatment is given if conservative methods show low efficacy and includes arthroscopic resection, which has been shown to be an effective and safe option [4].

Despite the significant clinical relevance [4, 5], precise and detailed morphometric parameters of the plica and its surrounding structures have yet to be analyzed in asymptomatic patients. Furthermore, the relation between the elbow plica and the surrounding anatomical landmarks has also not been thoroughly studied. Furthermore, there is still no consensus on the prevalence of the plica in asymptomatic group of people. Moreover, the literature lacks data concerning the morphology of synovial plica with respect to age and sex. Establishing these features is clinically significant to provide physicians with a set of statistically significant, repeatable, and easy-to-evaluate parameters. Those measurements can be referential to the ones gathered from symptomatic patients during the examination and therefore can direct the path to a successful diagnosis and minimize the chance of misdiagnosing the problem. Therefore, the objective of the present study was to provide morphometric properties of the synovial plica of the elbow and its relation to surrounding structures in asymptomatic patients. For this purpose, magnetic resonance images of the elbow of 216 patients were analyzed in detail.

## Materials and methods

### Study group

A retrospective study was conducted to establish the morphometric characteristics of the synovial plica of the elbow. The results of 216 consecutive patients, who underwent magnetic resonance imaging (MRI) of an elbow, were analyzed in the Department of Radiology of Jagiellonian University Medical College, Cracow, Poland, in October 2022. The condition for the inclusion of the MRI result into the present study was the certainty that the patient did not have any symptoms of lateral elbow pain at the time of the examination. For this purpose, patients’ medical histories were also analyzed and patients who had MRI examination performed due to lateral elbow pain were excluded. Additional exclusion criteria were established as follows: (1) previous elbow trauma affecting the plica and/or its close anatomical area, (2) significant artifacts that prevented accurate and precise imaging and/or measurement of the plica and/or its close anatomical area, (3) low quality and illegible images, and (4) absence of the plica in the studied material after the initial evaluation. Eventually, a total of 161 elbows met the required criteria.

### Results acquisition

All examinations were performed using 3.0 Tesla MRI scanner (Achieva, Philips Healthcare, Amsterdam, The Netherlands). To the further analysis, only T1-weighted and STIR (short tau inversion recovery) sequences were included. Detailed scan parameters were the following: (1) for T1-weighted turbo spin echo (TSE) sequence—TR 500 ms, TE 14 ms, flip angle 90, NEX 1, slice thickness 3 mm, matrix 560 × 560, FOV 240 × 240 × 71, (2) for STIR TSE sequence—TR 5239 ms, TE 30 ms, inversion time 190 ms, flip angle 90, NEX 2, slice thickness 3 mm, matrix 400 × 400, FOV 240 × 240 × 71.

The MRIs were analyzed on a dedicated workstation in the Anatomical Department of Jagiellonian University Medical College, Cracow, Poland. To ensure the highest possible quality of the visualizations and measurements and minimize potential bias, Materialise Mimics Medical version 22.0 software (Materialise NV, Leuven, Belgium) was used. 3-dimensional (3D) reconstructions of each scan were developed, employing a set of settings adjusted to each scan.

### Evaluation and measurements

Each plica and its close anatomical area were fully visualized at the beginning of each evaluation. Subsequently, a set of measurements was executed on each elbow as the following: (1) width of the plica, measured at its widest point, (2) length of the plica, measured at its longest diamension, (3) width of the radiohumeral joint gap, measured at the widest point of the plica, (4) width of the radiohumeral joint gap, measured at the middle of the radius articular surface, (5) width of the radiohumeral joint gap, measured at the medial end of the joint, (6) the distance from the edge of the radiohumeral joint to the plicas’ apex, (7) the length of the radius articular surface in the radiohumeral joint, (8) the length of the ulnar articular surface in the ulnohumeral joint, (9) the length of the common radio-ulnar articular surface in the proximal radio-ulnar joint, (10) the distance from the plicas’ edge to the apex of the proximal radio-ulnar articular surface, (11) the length of the radius head, (12) the thickness of the humerus, measured at its widest point, (13) the distance from the most lateral point of the radius head to the plicas’ edge, (14) the distance from the most lateral point of the lateral epicondyle of the humerus to the plicas’ edge, (15) plicas’ contact area with the humerus, (16) plicas’ contact area with the radius, (17) the length of the lateral edge of the radiohumeral joint, (18) the shortest distance from the plicas’ edge to the radial nerve bifurcation point, (19) the shortest distance from the plicas’ edge to the extensor carpi radialis longus (insertion), (20) the shortest distance from the plicas’ edge to the extensor carpi radialis brevis (insertion), (21) the shortest distance from the plicas’ edge to the extensor digitorum (insertion). In order to maintain objectivity and repeatability of the measurements, the same pattern was followed while establishing distances between structures. Each measurement was obtained twice by two independent researchers with radiological experience (surgeon and radiologist), and a mean value was established afterward. For radiological measurements, the interobserver and intraobserver reliability were calculated based on ten randomly selected patients using the reliability statistics of the interclass correlation (ICC). Both ICCs coefficients presented excellent values (ICC>0.95).

**Fig. 1:**
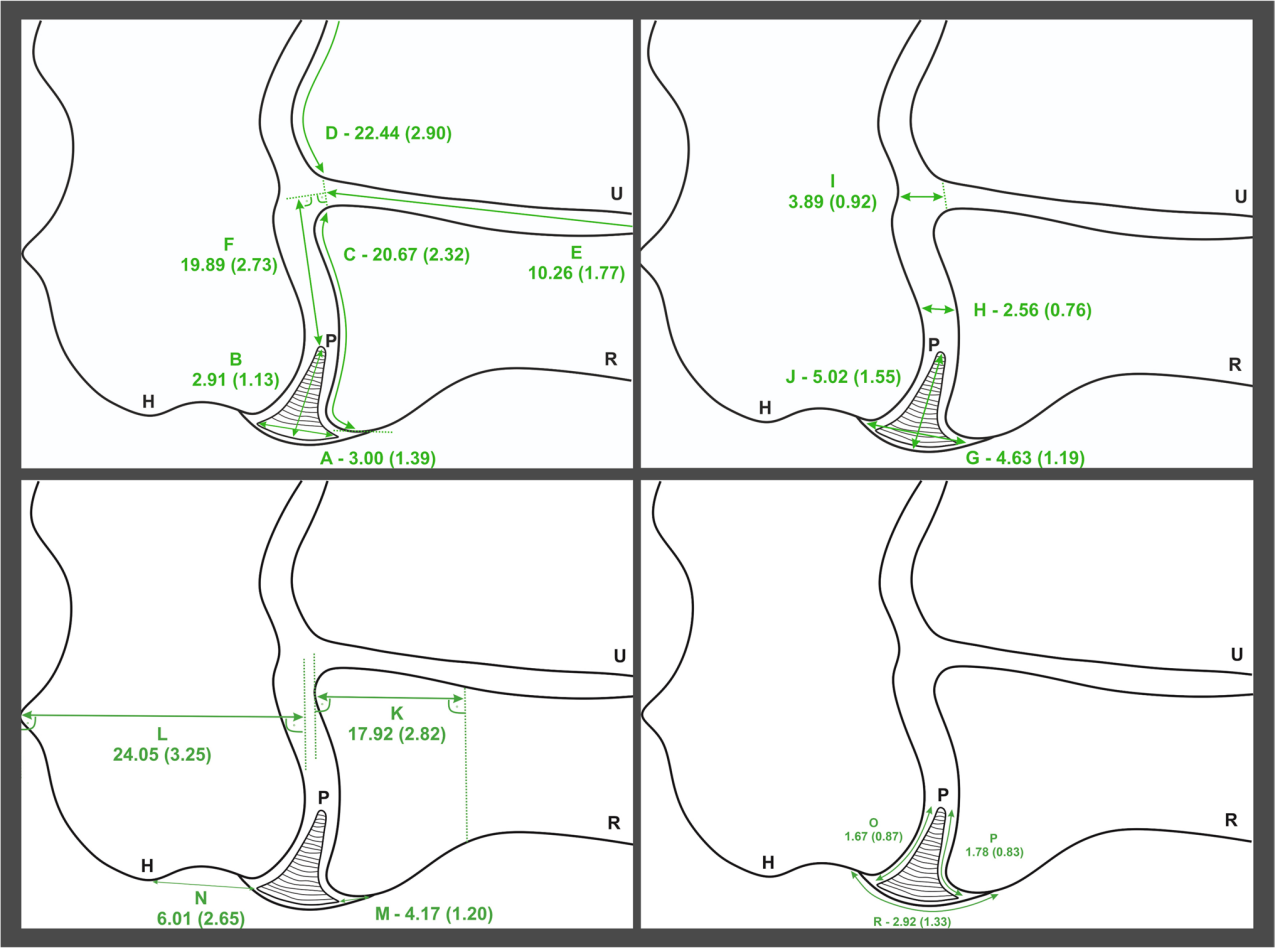
Scheme, illustrating some of the measurements conducted in this study. The mean results of the measurements in each category, with standard deviations in the brackets, were given next to the reference letters. All of the mentioned results are in millimeters. P - Plica. H - Humerus. R - Radius. U - Ulna. A - Plica - Width. B - Plica - Length. C - Articular Surface (Radius) - Length. D - Articular Surface (Ulna) - Length. E - Articular Surface (Proximal Radio-Ulnar) - Length. F - Distance from Plicas' edge to the apex of the proximal radio-ular articular surface. G - Joint Gap - Width (Plica). H - Joint Gap - Width (Middle). I - Joint Gap - Width (Medial End). J - Distance from the edge of the radiohumeral joint to the plicas’ apex. K - Radius Head - Length. L - Humerus - Thickness. M - Distance from the most lateral point of the Radius Head to the Plicas' Edge. N - Distance from the most lateral point of the Lateral Epicondyle of the Humerus to the Plicas' edge. O - Plicas' contact area with the Humerus. P - Plicas' contact area with the Radius. R - Edge of the joint – Length

**Fig. 2:**
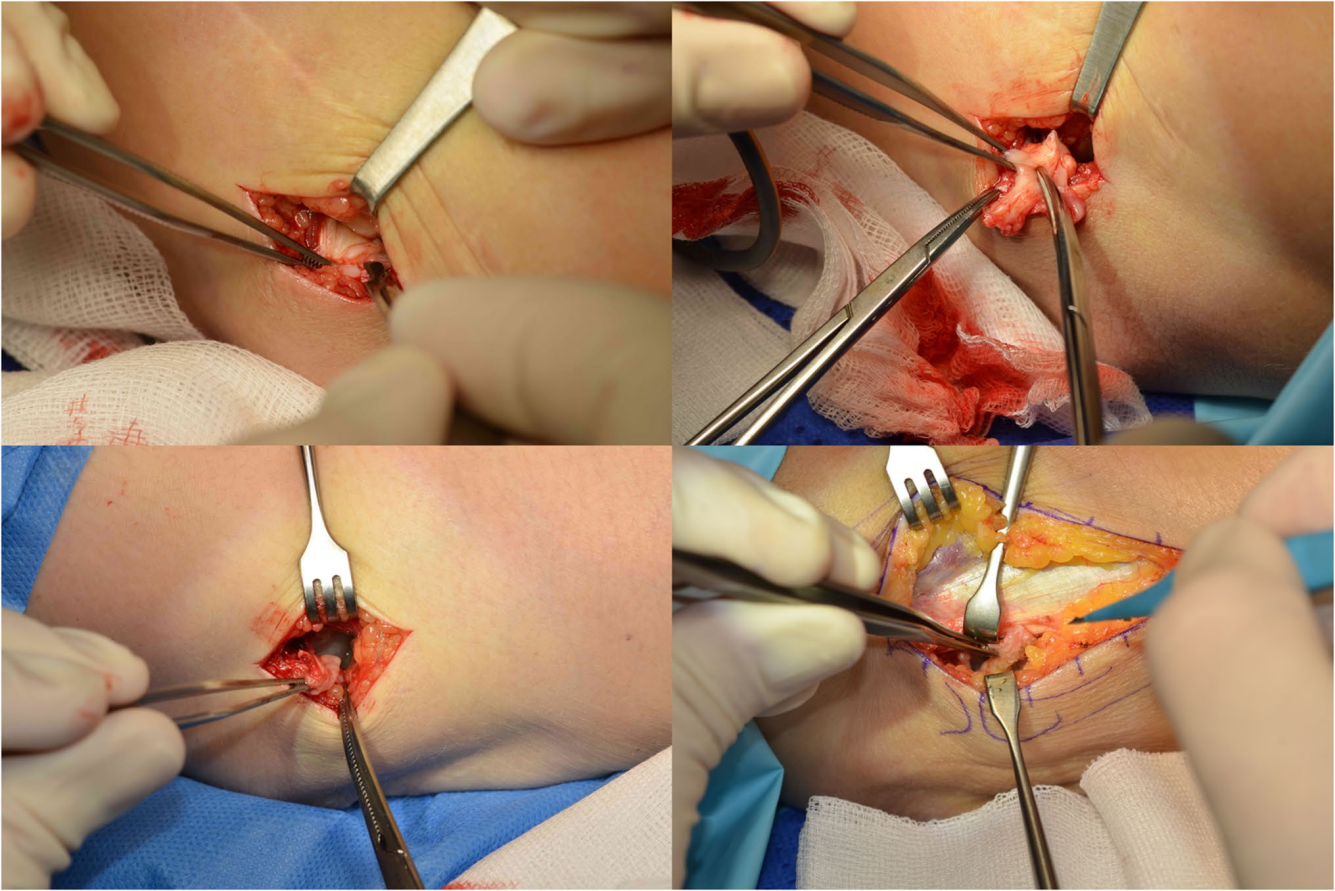
Intra-operative views of the enlarged synovial plica of the elbow

**Fig. 3:**
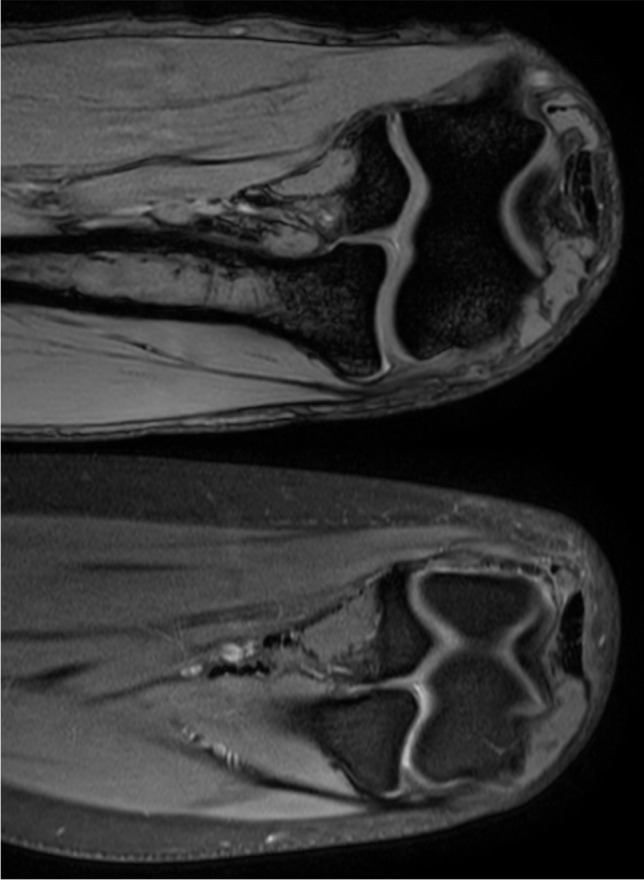
Views from the MRI scans of the synovial plicae of the elbow and their close anatomical areas

The measurement methods used in this study are presented in Fig. 1. The intra-operative view of the enlarged synovial plica of the elbow can be found in Fig. 2. Views of the synovial plicae of the elbow and their close anatomical areas from the MRI scans can be found in Fig. 3.

### Statistical analysis

Statistical analysis was performed with STATISTICA v13.1 (StatSoft Inc., Tulsa, OK, USA). The frequencies and percentages presented qualitative features. The Shapiro-Wilk test was used to assess the normal distribution. Quantitative characteristics were presented by medians and upper and lower confidence intervals (LCI, UCI), as well as means and standard deviation (SD), depending on verified normality of the data. Statistical significance was defined as *p* < 0.05. Mann-Whitney and Wilcoxon signed rank tests were used to establish potential differences between the groups. Spearman’s rank correlation coefficient was used to determine possible correlations between the parameters.

## Results

### Baseline characteristics

Plica was found in a total of 161 of 216 elbows (74.5%). MRI results were obtained from patients between 18 and 67 years of age, with a mean age of 37.44 years (SD: 12.34). Out of those, 53 (32.9%) were from women and 108 (67.1%) were from men.

### Measurements results

The mean width of the plica was set to be 3.00 mm (SD: 1.39). The mean length of the plica was established at 2.91 mm (SD: 1.13). The mean width of the radiohumeral joint gap, measured at the widest point of the plica was set to be 4.63 mm (SD: 1.19). The mean width of the radiohumeral joint gap, measured at the middle of the radial articular surface was established at 2.56 mm (SD: 0.76). The mean width of the radiohumeral joint gap, measured at the medial end of the joint was set to be 3.89 mm (SD: 0.92). The mean distance from the edge of the radiohumeral joint to the plicas’ apex was established at 5.02 mm (SD: 1.55). The mean length of the radius articular surface in the radiohumeral joint was set to be 20.67 mm (SD: 2.32). The mean length of the ulnar articular surface in the ulnohumeral joint was established at 22.44 mm (SD: 2.90). The mean length of the common radio-ulnar articular surface in the proximal radio-ulnar joint was set to be 10.26 mm (SD: 1.77). All of the results mentioned above and more detailed ones can be found in Table 1.

**Table 1:** Overall results of the measurements. LCI – lower confidence interval. All metrical results are in millimeters [mm]. UCI – upper confidence interval. Min – minimal value. Max – maximal value. SD – standard deviation

Category	Mean	SD	Median	LCI	UCI	Min	Max
Age	37.44	12.34	38.00	29.00	45.00	18.00	67.00
Plica - Width	3.00	1.39	2.67	2.06	3.48	1.16	9.81
Plica - Length	2.91	1.13	2.68	2.16	3.33	1.30	9.78
Joint Gap - Width (Plica)	4.63	1.19	4.43	3.83	5.11	2.31	9.05
Joint Gap - Width (Middle)	2.56	0.76	2.52	2.08	2.86	1.21	6.27
Joint Gap - Width (Medial End)	3.89	0.92	3.79	3.19	4.59	2.00	6.04
Distance from the edge of the radiohumeral joint to the plicas’ apex	5.02	1.55	4.75	3.93	5.87	2.19	12.16
Articular Surface (Radius) - Length	20.67	2.32	20.59	19.05	22.57	14.41	26.96
Articular Surface (Ulnar) - Length	22.44	2.90	22.31	20.66	24.24	15.51	31.73
Articular Surface (Proximal Radio-Ulnar) - Length	10.26	1.77	10.11	9.09	11.31	6.67	16.72
Distance from Plicas' edge to the apex of proximal radio-ulnar articular surface	19.89	2.73	19.86	18.42	21.65	3.73	25.52
Radius Head - Length	17.92	2.82	17.85	15.92	19.74	11.83	28.76
Humerus - Thickness	24.05	3.25	24.19	22.29	25.73	4.94	32.53
Distance from the most lateral point of the Radius Head to the Plicas' edge	4.17	1.20	4.09	3.50	4.64	1.79	13.55
Distance from the most lateral point of the Lateral Epicondyle of the Humerus to the Plicas' edge	6.01	2.65	5.54	4.26	6.57	2.49	22.54
Plicas' contact area with the Humerus	1.67	0.87	1.48	1.04	2.03	0.33	4.92
Plicas' contact area with the Radius	1.78	0.83	1.64	1.20	2.11	0.29	5.56
Edge of the Joint - Length	2.92	1.33	2.62	2.11	3.48	0.83	12.26
Shortest distance from the plicas' lateral edge to the Radial Nerve bifurcation point	32.13	6.50	31.45	27.70	35.21	19.64	65.21
Shortest distance from the plicas' lateral edge to the Extensor Carpi Radialis Longus	29.28	6.39	28.31	24.95	32.48	16.45	58.54
Shortest distance from the plicas' lateral edge to the Extensor Carpi Radialis Brevis	20.96	3.65	20.70	18.71	22.89	12.13	34.98
Shortest distance from the plicas' lateral edge to the Extensor Digitorum	16.19	3.73	16.16	13.57	18.28	6.86	31.26

### Sexual dimorphism

An analysis of sexual dimorphism was also included. Regarding all categories mentioned in Table 1, potential differences between men and women were analyzed. The measurement results obtained from male cases were significantly higher in 17 of 21 categories (*p* < 0.05). All the results and more detailed descriptions of sex differences are presented in Table 2.

**Table 2:**
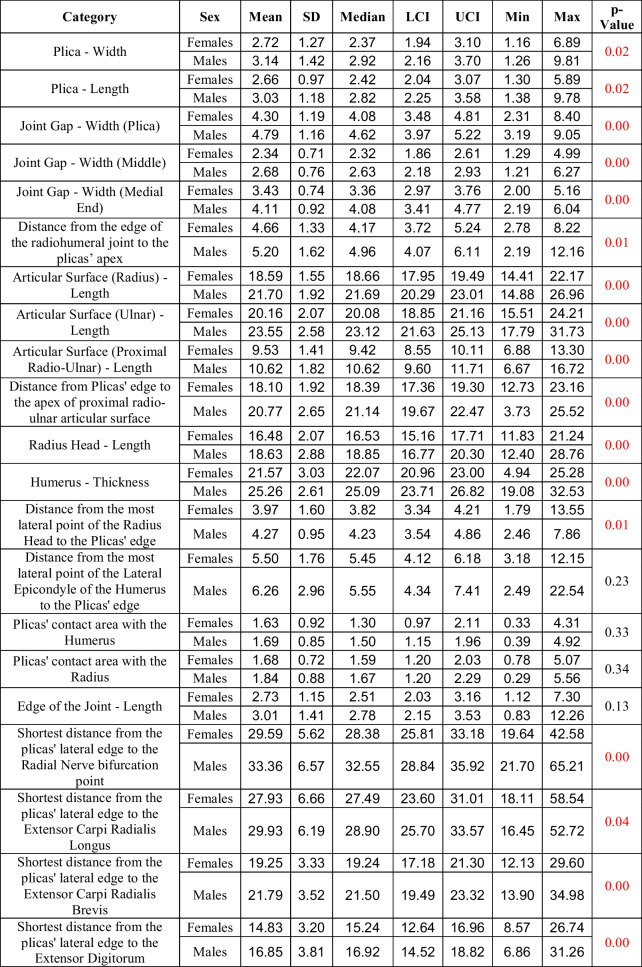
Results of the measurements with respect to patients’ sex. All metrical results are in millimeters [mm]. Highlighted in red are those in which the p-value was less than 0.05. LCI – lower confidence interval. UCI – upper confidence interval. Min – minimal value. Max – maximal value. SD – standard deviation

### Associations between parameters

Potential correlations were analyzed for each of the categories and age. The only parameter that was statistically significantly correlated with age was the length of the edge of the radiohumeral joint (*R* = −0.16 ; *p* = 0.01). However, some categories correlate with each other. The associations between the categories are summarized in Table 3.

**Table 3:**
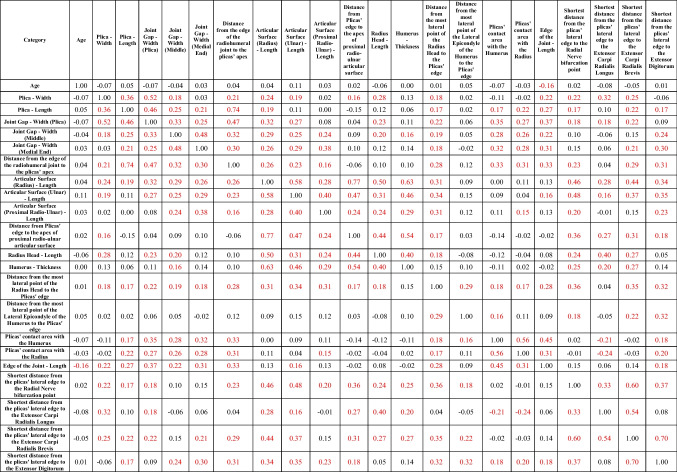
Gathers the *R* values obtained in the correlation analysis between categories. Highlighted in red are those in which the *p* value was smaller than 0.05

## Discussion

The overall prevalence of the synovial plica of the elbow has been discussed by other studies in the past. The prevalence of the plica has been analyzed in both asymptomatic and symptomatic patients, in cadaveric and/or radiological studies [2, 3, 6–10]. Duparc et al. conducted a cadaveric study about the anatomical and histological features of the plica [2]. In the study, the plica had a high frequency of 86%. Choi et al. analyzed the elbow plica in asymptomatic and symptomatic subjects [8]. Their study consisted of 50 asymptomatic elbows (the asymptomatic group) and 14 elbows with confirmed posterolateral plica syndrome by arthroscopy (the plica group). They found that the plica group showed a higher prevalence on conventional magnetic resonance imaging compared to asymptomatic subjects [8]. However, one significant limitation of that study is the low number of subjects used. Our study, which is based on 216 elbows of asymptomatic patients, is the largest one concerning the plica in the available literature. The present analysis shows that the overall prevalence of synovial plica of the elbow in asymptomatic patients was 74.5% (161 of 216 elbows). This further proves the consistency of the plica, making it a prevalent anatomical entity.

The elbow plica is contiguous with the capsule-ligament complex projected into the free space of the joint. However, their defining location has been a subject of confusion. Cerezal et al. and Isogai et al. presented a more detailed description of the location of the plica [1, 3]; the anterior fold, a thin part of the radiohumeral synovial fold, having a frequency of 67% to 100%; the lateral fold, thin, crescent or meniscoid shape, present in 5 to 20%; the posterior fold, between the lesser and greater sigmoid cavities and radiohumeral surfaces, merges with the lateral fold anteriorly and the lateral olecranon fold, present in 86 to 100%; the lateral olecranon fold, on the lateral margin of the olecranon, under the anconeus muscle, present in 28 to 33% of the cases; and lastly, the circumferential fold, described as a continuous plate, combing the anterior and posterior folds, present in 2 to 12% [1, 3, 7].

Our analysis of plicae in an asymptomatic group of individuals shows that the mean width (or thickness, depending on the nomenclature used) of the synovial fold is 3.00 mm (SD: 1.39). This would contradict the results of the study by Awaya et al. [10], where the thickness in asymptomatic patients was less than 2.0 mm, and in symptomatic patients the average thickness was 3.1 mm. The authors stated that thickness of >3.0 mm could indicate that the patient could have synovial fold syndrome. However, after a comparison of our measurement results from the asymptomatic group with the measurement results from symptomatic patients from the said study, it has been established that there are no statistically significant differences between those two groups (*p* = 0.88). Similar conclusions appear after a comparison of our results with the results of the study by Luzuriaga et al. [11]. In their study, the mean thickness of the plica in symptomatic patients was 2.5 mm. Thus, again, no statistically significant difference can be observed (*p* = 0.50). We did not compare the results of the current study with the results of Celikyay et al. [12] research because their study consisted of plica measurements taken from patients with osteoarthritis. However, the mean thickness of the synovial fold in symptomatic patients with osteoarthritis was set at 1.42 mm. The absence of statistically significant differences may be due to the small number of symptomatic patients evaluated in the said studies. However, simultaneously, it may suggest that thickness (or width, depending on the nomenclature used) may not be an appropriate parameter for evaluation of plica in daily clinical practice as it may lead to heterogeneous conclusions. However, the authors of the current study suggest evaluating more of the plica’s parameters in daily clinical practice. The most detailed evaluation seems to be based on the resultant of plicas’ contact areas with the humerus and radius, width of the joint gaps, length of the edge of the joint and plicas length and width. Taking into account the results of the current study, physicians can compare the results obtained during the examination and establish an overall and most holistic overview of the patient’s plica. Additionally, expansion of the synovial fold can proceed disproportionately in all dimensions; thus comparing a number of them seems to be the most reliable option when suspecting a pathology of the synovial fold.

The present study is the first to provide detailed measurements of the relations of the synovial plica with other anatomical structures of the elbow. We measured the distances from the lateral edge of the plicae to numerous structures, such as the point of bifurcation of the radial nerve and the extensors, mainly the extensor carpi radialis longus and extensor digitorum, among others. Furthermore, the contact area between the plica and the humerus and radius was also measured. In future research, these parameters should be measured in symptomatic patients because they could potentially provide physicians with more parameters for the diagnosis of symptomatic synovial plica syndrome. Physicians should also take into account synovial fold syndrome when diagnosing other lateral elbow conditions such as lateral epicondylitis (most commonly known as tennis elbow), oppression of the radial and/or posterior interosseous nerve, or snapping of the triceps tendon [13, 14]. Differences in symptoms of these conditions may be very detailed and commonly imperceptible. This makes the differentiation of the source of lateral elbow pain very complicated, which eventually leads to a misdiagnosis and unsuccessful treatment. Awareness of the plica and its clinical relevance should be raised among physicians, especially surgeons, orthopedists, and radiologists dealing with lateral elbow pain in daily practice.

The present study is the first to examine the potential differences between women and men in plica parameters and to establish potential correlations between these measurements and the age of the patients. It may be concluded that age should not necessarily be taken into account when examining the synovial fold syndrome, as none of the parameters, besides the length of the edge of the joint, statistically significantly correlated with age. However, the statistically significant difference between women and men was established in most of the categories. Therefore, when physicians compare their measurements with the results presented in our study, it is crucial to make the comparison with the same gender group as the patients’, rather than with the overall results. Furthermore, a comparison of symptomatic and asymptomatic plicae in future studies should also be performed with respect to the sex of the patients.

This study is not without limitations. The authors did not compare the results obtained using magnetic resonance imaging with other imaging methods, such as ultrasound, which is used more frequently in daily clinical practice. Additionally, more measurements should be evaluated in further studies, in other positions of the elbow, as the plica may change some of its measurements while moving. The authors believe that a meta-analysis and/or more primary studies should be performed in the future to provide a homogeneous conclusion on plica thickness in the diagnosis of synovial fold syndrome.

## Conclusion

The synovial plica of the elbow is a clinically relevant anatomical structure. Analyzing the morphometric parameters of the synovial plica is necessary to properly evaluate synovial plica syndrome, which can commonly be confused with other sources of lateral elbow pain such as tennis elbow, oppression of the radial and/or posterior interosseous nerve, or snapping of the triceps tendon. The authors suggest that the thickness of the plica may not be the golden diagnostic feature as there are no statistically significant differences in this parameter between symptomatic and asymptomatic patients. The most detailed evaluation appears to be based on the measurements of plicas’ contact areas with the humerus and radius, width of the joint gaps, length of the edge of the joint and plicas length and width. Therefore, when physicians compare their measurements with the results presented in our study, it is crucial to make the comparison with the same gender group as the patients’, rather than with the overall results. A precise and accurate diagnosis of synovial fold syndrome and/or differentiation from other sources of lateral elbow pain must be performed, as the surgical treatment, even if performed properly, will be unsuccessful because of a misdiagnosed source of pain.

## Data Availability

The data that support the findings of this study are available from the corresponding author, upon reasonable request.
